# Loss to follow-up among children and adolescents growing up with HIV infection: age really matters

**DOI:** 10.7448/IAS.20.1.21737

**Published:** 2017-07-17

**Authors:** Katharina Kranzer, John Bradley, Joseph Musaazi, Mary Nyathi, Hilary Gunguwo, Wedu Ndebele, Mark Dixon, Mbongeni Ndhlovu, Andrea Rehman, Palwasha Khan, Florian Vogt, Tsitsi Apollo, Rashida Abbas Ferrand

**Affiliations:** ^a^ Department of Infectious Disease Epidemiology, London School of Hygiene & Tropical Medicine, London, UK; ^b^ National and Supranational Reference Laboratory, Leibniz Research Centre, Schleswig-Holstein, Germany; ^c^ Infectious Disease Institute, Kampala, Uganda; ^d^ Department of Medicine, Mpilo Central Hospital, Bulawayo, Zimbabwe; ^e^ Department of Medicine, National University of Science and Technology, Bulawayo, Zimbabwe; ^f^ Institute of Tropical Medicine, Antwerp, Belgium; ^g^ AIDS and TB Unit, Ministry of Health and Child Welfare, Harare, Zimbabwe; ^h^ Department of Medicine, University of Zimbabwe, Harare, Zimbabwe; ^i^ Department of Clinical Research, London School of Hygiene & Tropical Medicine, London, UK; ^j^ Biomedical Research and Training Institute, Harare, Zimbabwe

**Keywords:** transition, adolescent, HIV, Africa, lost-to-follow-up

## Abstract

**Introduction**: Globally, increasing numbers of HIV-infected children are reaching adolescence due to antiretroviral therapy (ART). We investigated rates of loss-to-follow-up (LTFU) from HIV care services among children as they transition from childhood through adolescence.

**Methods**: Individuals aged 5–19 years initiated on ART in a public-sector HIV clinic in Bulawayo, Zimbabwe, between 2005 and 2009 were included in a retrospective cohort study. Participants were categorized into narrow age-bands namely: 5–9 (children), 10–14 (young adolescents) and 15–19 (older adolescents). The effect of age at ART initiation, current age (using a time-updated Lexis expansion) and transitioning from one age group to the next on LTFU was estimated using Poisson regression.

**Results**: Of 2273 participants, 1013, 875 and 385 initiated ART aged 5–9, 10–14 and 15–19 years, respectively. Unlike those starting ART as children, individuals starting ART as young adolescents had higher LTFU rates after moving to the older adolescent age-band (Adjusted rate ratio (ARR) 1.54; 95% CI: 0.94–2.55) and similarly, older adolescents had higher LTFU rates after transitioning to being young adults (ARR 1.79; 95% CI: 1.05–3.07). In older adolescents, the LTFU rate among those who started ART in that age-band was higher compared to the rate among those starting ART at a younger age (ARR = 1.70; 95% CI: 1.05, 2.77). This however did not hold true for other age-groups.

**Conclusions**: Adolescents had higher rates of LTFU compared to other age-groups, with older adolescents at particularly high risk in all analyses. Age-updated analyses that examine movement across narrow age-bands are paramount in understanding how developmental heterogeneity in children affects HIV outcomes.

## Introduction

In 2015, there were 2 million adolescents aged 10–19 living with HIV worldwide [1]. The remarkable scale-up of paediatric antiretroviral therapy (ART) globally has resulted in increasing numbers of infants infected with HIV surviving to adolescence and beyond; and the number of HIV-infected adolescents is projected to continue increasing for at least a decade [2]. The scale-up of prevention of mother-to-child HIV transmission programmes started in 2005 resulting in a 75% decrease in new paediatric HIV infections globally [1]. Despite this success, an estimated 150,000 children (aged 0–14 years) were newly infected with HIV in 2015, nearly 85% of them in sub-Saharan Africa. Many of these children will present to healthcare services in adolescence [3].

Despite the significant disease burden in this age group, adolescents living with HIV fall through the gaps of poorly coordinated health systems and HIV programmes that have focused on adults, infants and young children. Research studies and programmes often exclude adolescents or group them with children and adults (e.g. 0–15 years, 15+ years) [4]. If age-stratified analyses are conducted, individuals are usually categorized by age at ART initiation, an approach widely used for adult ART cohorts [4]. Most cohort analyses of HIV-infected children and adolescents do not consider the significant heterogeneity in development across this age group or the impact of the phase of rapid physical and psychosocial development during adolescence. Therefore, longitudinal analyses investigating HIV care outcomes that stratify children based on age at ART initiation will fail to account for the impact of developmental changes on health outcomes.

We conducted a cohort study of older children, adolescents and young adults on ART in Zimbabwe. The study aimed to investigate outcomes using narrow five-year age-bands and specifically investigated the effect of current age and moving into the next age-band on loss to follow-up (LTFU) rates.

## Methods

### Study setting and population

Zimbabwe experienced an early onset HIV epidemic with antenatal HIV prevalence peaking at 30% in 1997. The HIV incidence has subsequently declined but HIV prevalence remains high (HIV prevalence among adults in 2015 at 15%). During the period of this study, Zimbabwe experienced massive hyperinflation, a shrinking economy and food shortages.

Participants were patients who initiated ART aged 5–19 years between 1 January 2005 and 31 December 2009 at the Mpilo Central Hospital HIV clinic in Bulawayo, the second largest city in Zimbabwe. The Mpilo clinic started to provide ART in April 2004, the first public sector facility to do so in Zimbabwe. It has therefore accumulated a large cohort of adults and paediatric patients on ART with long periods of follow-up. Details about the operation of the clinic are given elsewhere [5], but briefly HIV care at the clinic was provided by the government of Zimbabwe with several partners including Medecins Sans Frontieres. HIV treatment was provided in accordance with Zimbabwean National Guidelines, with individuals eligible for ART if they had a CD4^+^ cell count less than 200 cells/μl and/or WHO stage III or IV HIV disease. HIV care was provided free of charge. Each clinic visit and the individual’s next scheduled visit were routinely recorded using FUCHIA software (Epicentre, Paris, France). ART refill visits were scheduled monthly for the first three months on ART and three monthly thereafter. Patients were seen by nurses or doctors before they were sent to pick up the drugs. Systematic tracing of defaulting ART patients, defined as having missed a scheduled appointment by more than two months, was done by community volunteers through home visits and telephone calls. Community volunteers also recorded death. In addition, mortality data were obtained through notification by family and through death register review. Longitudinal patient data were analysed from time of ART initiation up to 31 December 2010 to allow at least one year of follow-up.

### Data analysis

Data were analysed using STATA version 14 (STATA corporation, Texas, USA). The primary endpoint was time to LTFU after ART initiation. LTFU was defined as being late for an appointment for more than 60 days (i.e. the date of the next scheduled appointment plus 60 days) at the date of censoring (31 December 2010). The date of LTFU was set at the date of the next appointment. Death and transfer outs were treated as censoring events. Baseline characteristics stratified by age group were described by frequencies for categorical variables. CD4 count at initiation of ART was summarized as medians and inter-quartile range.

Age was the explanatory variable of interest. Individuals aged 5–9 years, 10–14 years, 15–19 years and 20–24 years are referred to as older children, young adolescents, older adolescents and young adults, respectively [6]. Poisson models were used to estimate the crude LTFU rate. A Lexis expansion was performed to control for calendar year and time on ART (<6 months, 6months to <1 year and ≥1 year). Analyses were adjusted for sex, calendar time and time on ART. Rate ratios were calculated to investigate the effect of age at ART initiation on LTFU. A Lexis expansion was also performed to estimate the effect of current age on rate of LTFU (age-updated analysis). LTFU rates were calculated before and after children transferred to young adolescence, young adolescents transferred to older adolescence and older adolescents transferred to young adulthood. Rates of LTFU were compared in those who started ART in their current age group to those who started ART in the previous age group.

### Ethics

The requirement for a formal ethical review and individual consent from patients to use clinical data was waived by the Medical Research Council of Zimbabwe as anonymized data were used and no personal identification information was collected.

## Results

### Characteristics of the cohort

A total of 2273 individuals were included in the analysis. Of these, 1013 (37%), 875 (33%) and 385 (16%) initiated ART at the age of 5–9, 10–14 and 15–19 years, respectively (Table 1). A total of 52% of the individuals initiating ART aged 5–9 years (older children) moved to the young adolescent group before the end of follow-up (Supplementary Figure 1); 45% and 27% of those starting ART as young adolescents and older adolescents moved to the next age-group, that is, to older adolescence and young adulthood, respectively, over the follow-up period. The number of males and females were similar in all age-bands, except for the older adolescents with slightly more females (56%) (Table 1). A total of 65% of the cohort had WHO Stage III/IV HIV disease at ART initiation and median CD4 count at ART initiation was 211 cells/μl.

**Table 1.** Description of participants by baseline age group at ART initiation

Characteristics	5–9 years	10–14 years	15–19 years	Total
Number	1013	875	385	2273
Female, *n* (%)	461 (46)	430 (49)	215 (56)	1106 (49)
WHO disease stage 3 or 4, *n* (%)	657 (65)	577 (66)	239 (62)	1473 (65)
CD4 cells count/mL, median (IQR)	260 (114, 417) *(N = 553)*	191 (84, 330) *(N = 429)*	163 (33, 255) *(N = 165)*	211 (90, 379) *(N = 1147)*
Year of ART start, *n* (%)				
2005	107 (11)	85 (10)	37 (10)	229 (10)
2006	171 (17)	131 (15)	32 (8)	334 (15)
2007	226 (22)	194 (22)	75 (19)	495 (22)
2008	259 (26)	215 (25)	95 (25)	569 (25)
2009	250 (25)	250 (29)	146 (38)	646 (28)
Number of person years of follow-up	2624	2153	794	5571
Follow-up time in years per subject, median (IQR)	2.45 (1.44, 3.64)	2.30 (1.34, 3.43)	1.79 (1.15, 2.74)	2.27 (1.30, 3.44)

*IQR = Inter-Quartile Range; ART = antiretroviral therapy*.

### Rates of loss to follow-up

The cohort was observed for 5571 person years, with 194 (8.4%) individuals’ LTFU, resulting in an overall LTFU rate of 4.92 (95% CI: 4.37, 5.54) per 100 person-years (PY). The median follow-up time before LTFU was 1.01 years (IQR 0.41–2.04), and the median age at LTFU was 13 years (IQR 9–18). The cohort grew larger with time: there were 229 participants in care in 2005 and 2033 in 2009 (Table 2).

**Table 2.** Rates of LTFU by calendar year

Year	Number in care during the calendar year	Follow-up time (person years)	Number lost to follow-up	Rate (95% CI) per 100 person years	Crude RR	Adjusted RR*
**2005**	229	107	3	2.81 (0.91, 8.70)	1	1
**2006**	539	329	14	4.25 (2.52, 7.18)	1.51 (0.43, 5.27)	1.90 (0.54, 86.64)
**2007**	978	709	26	3.67 (2.50, 5.38)	1.31 (0.40, 4.32)	1.73 (0.52, 5.76)
**2008**	1497	1156	70	6.05 (4.79, 7.65)	2.16 (0.68, 6.85)	3.07 (0.96, 9.84)
**2009**	2033	1627	81	4.97(4.00, 6.19)	1.77 (0.56, 5.61)	2.54 (0.79, 8.15)
*p*-Value					0.169	0.046

*adjusted for sex, current age and time on ART. *LTFU = loss-to-follow-up*.

### The effect of current age (age-updated) and age at ART initiation

The rate of LTFU per 100 PY in those who started ART aged 5–9, 10–14 and 15–19 years was 4.08 (95% CI 3.37, 4.93), 3.86 (95% CI 3.11, 4.78) and 10.58 (95% CI 8.55, 13.11), respectively. The rate of LTFU per 100 PY in those currently aged 5–9, 10–14, 15–19 and 20–24 years was 5.07 (95% CI 4.10, 6.26), 2.97 (95% CI 2.37, 3.73), 7.39 (95% CI 6.03, 9.06) and 16.91 (96% CI 11.03, 25.94), respectively (*p* < 0.001) (Table 3). The salient difference in findings between these two analyses is that although young adolescents have statistically significant lower rates of LTFU than children (Adjusted Rate Ratio (ARR) 0.63; 95% CI 0.46, 0.86), those who start ART as young adolescents have similar rates of LTFU (ARR 0.93; 95% CI 0.70, 1.24) to those who start ART as children.

**Table 3.** Unadjusted and adjusted rate ratios estimates of LTFU comparing young children to other age groups

	Effect of current age			Effect of age at ART start		
Age group	Rate (95% CI) per 100 Person Years	Crude RR	Adjusted* RR	Rate (95% CI) per 100 Person Years	Crude RR	Adjusted* RR
Older children (5–9 years)	5.07 (4.10, 6.26)	1	1	4.08 (3.37, 4.93)	1	1
Young adolescents (10–14 years)	2.97 (2.37, 3.73)	0.57 (0.43, 0.80)	0.63 (0.46, 0.86)	3.86 (3.11, 4.78)	0.95 (0.71, 1.26)	0.93 (0.70, 1.24)
Older adolescents (15–19 years)	7.39 (6.03, 9.06)	1.46 (1.09, 1.96)	1.58 (1.17, 2.13)	10.58 (8.55, 13.11)	2.60 (1.95, 3.45)	2.43 (1.82, 3.24)
Young adults (20–24 years)	16.91 (11.03, 25.94)	3.34 (2.07, 5.38)	3.94 (2.41, 6.46)	-	-	-
*p*-Value*		<0.001	<0.001		<0.001	<0.001

*adjusted for duration of ART, sex and calendar year. *LTFU = loss-to-follow-up*.

### The effect of transitioning to the next age group

The rate of LTFU in individuals who started ART as young children reduced after moving to the young adolescent age group, ARR = 0.63 (95% CI 0.37, 1.08) (Table 4). In those who started ART as young adolescents, rates of LTFU were significantly higher after moving to being older adolescents, ARR = 1.54 (95% CI 0.94, 2.55). Similarly, those who started ART as older adolescents had higher rates of LTFU after transitioning to being young adults, ARR = 1.79 (95% CI 1.05, 3.07).

**Table 4.** Rates of LTFU before and after transition to next age-band

Age at ART start	Rate before transition to next age-band (per 100 person years), (95% CI)	Rate after transition to next age-band (per 100 person years), (95% CI)	RR (95% CI), *p*-value	Adjusted* RR (95% CI), *p*-value
**Older children** **(5–9 years)**	5.07 (4.10, 6.26)	2.27 (1.48, 3.48)	0.45 (0.28, 0.72), *p* = 0.001	0.63 (0.37, 1.08), *p* = 0.094
**Young adolescents (10–14 years)**	3.39 (2.59, 4.43)	4.95 (3.44, 7.12)	1.46 (0.93, 2.30), *p* = 0.100	1.54 (0.94, 2.55), *p* = 0.089
**Older adolescents** **(15–19 years)**	9.54 (7.47, 12.19)	16.26 (10.49, 25.20)	1.70 (1.03, 2.81), *p* = 0.038	1.79 (1.05, 3.07), *p* = 0.033

*adjusted for time on ART, sex and calendar year. *LTFU = loss-to-follow-up*.

### The effect of age at ART initiation within each age group

In older adolescents, the rate of LTFU among those who started ART in that age-band was higher compared to the rate of LTFU in those who started ART at a younger age (ARR = 1.70 (95% CI 1.05, 2.77)) (Table 5). In young adolescents, however, there was no evidence that the rate of LTFU depended on the age of ART initiation; young adolescents initiated on ART as children had similar rates of LTFU (2.27 per 100 PY) compared to young adolescents starting ART as young adolescents (3.39 per 100 PY), ARR = 1.33 (95% CI 0.76, 2.32).

**Table 5.** Rates of loss to follow-up in different age groups according to when they started ART

	Loss to follow-up			
Current age	Rate in those who started ART in this age group (per 100 person years)	Rate in those who started ART in a younger age group (per 100 person years)	RR (95% CI), *p*-value	Adjusted* RR (95% CI), *p*-value
**Young adolescents** **(10–14 years)**	3.39 (2.59, 4.43)	2.27 (1.48, 3.48)	1.49 (0.90, 2.47), *p* = 0.121	1.33 (0.76, 2.32), *p* = 0.315
**Older adolescents** **(15–19 years)**	9.54 (7.47, 12.19)	4.95 (3.44, 7.12)	1.93 (1.24, 2.99), *p* = 0.003	1.70 (1.05, 2.77), *p* = 0.032

*adjusted for time on ART, sex and calendar year. ART = antiretroviral therapy.

## Discussion

The key finding of this cohort study is that there is a significantly increased risk of LTFU in adolescents. Older adolescents (15–19 year olds) appear particularly vulnerable. This age group showed an increased rate of LTFU in all analyses: those starting ART as young adolescents (10–14 years) had a 1.5 times higher rate of LTFU after moving into the 15–19 age group; adolescents starting ART aged 15–19 had an increased rate of LTFU compared to adolescents of the same age established on ART at an earlier age; and moving from adolescence into young adulthood increased the risk of LTFU by nearly twofold.

Chronic care services have long recognized problems with retention in care among chronically ill children during the transitional period between childhood to adulthood [7,8]. Data from high-income countries show that adolescents living with HIV are at increased risk of LTFU and poor adherence when reaching young adulthood. In the UK, young adults aged 15–24 years had an increased risk of LTFU compared to older adults [9]. A study from the US showed that the risk of advanced immunosuppression and detectable viraemia increased significantly as patients grew older notably at ages 15–19 and older [10]. These data are in line with experience from low-income countries where clinicians have reported problems with adherence and poor clinic retention among HIV-infected adolescents [11]. However, most cohort studies to date have failed to show an increased risk of LTFU among adolescents in low resource settings [4,5,12]. The discrepancies between anecdotal evidence and cohort analysis performed to date may be partly related to methodological issues [4].

Cohort studies and programmatic reporting have often lumped HIV-infected children and adolescents together [13,14]. Furthermore, analyses categorize individuals by the age of ART initiation rather than current age and do not consider the impact of the transition from childhood to adolescence and adulthood on care outcomes [4]. Unlike in adults, where behaviour and cognitive function are relatively stable across 5–10 year age-bands, adolescence is a critical phase of development accompanied by rapid physical, psychosocial and emotional changes, which influence health-related behaviour and outcomes [15,16]. Cognitive, social and psychological functioning is therefore not only extremely heterogeneous across the age range of 0–24 years, but is strongly associated with factors that may influence HIV care outcomes. For example, the stage of development influences the extent to which parents and guardians are involved in healthcare. Children are usually accompanied to clinic appointments by their parents or guardians who also facilitate adherence to medication. In contrast, adolescents are expected to take responsibility for their own health when they may not have fully acquired the skills required to manage a chronic illness [17,18]. Disclosure of HIV status to the child and adolescent depends upon their emotional maturity and their ability to process information appropriately, and disclosure status is strongly associated with outcomes [19,20]. Age also matters when it comes to navigating a complex social environment, as is often the case for children growing up with HIV. Peer relationships become highly influential as children approach adolescence [21]. Children and adolescents living with chronic medical conditions such as HIV often endure social isolation [22] and experience external as well as internalized stigma as a result of feeling different from others [23,24]. Social pressures to “fit in” may place this population at high risk for engaging in health risk behaviour including dropping out of HIV care.

The age-related rapid change of cognitive, psychosocial and psychomotor development calls for an analytic approach using narrow age-bands such as those used in our study. Data for children and adolescents have been analysed in a variety of ways categorizing children and adolescents into variably aggregated age-bands in comparison with adults, for example, adolescents (10–19 years) [25], children (<15 years) [14] and youth (15–24 years) [26]. Careful consideration to decide how best to categorize persons in the 0–24 year age group is needed. An optimized and standardized approach is required to minimize heterogeneity in findings. We propose the use of five year age-bands to separate older children (5–9 years), from young adolescents (10–14 years), older adolescents (15–19 years) and young adults (20–24 years) [12]. Furthermore, most analyses classify individuals into age-bands based on age at initiation of ART. It is only a matter of time before an individual moves into the next age-band but this is not considered in standard cohort analyses, which then results in misclassification. Age-updated analysis taking into account the current age of individual should therefore be the standard when conducting cohort analysis among children and adolescents.

The strength of the analysis is the number of children, adolescents and young adults included and the use of the data from a public-sector health setting, which makes the findings broadly generalizable. We utilized a novel approach to investigate outcomes using age-updated analyses that enables assessment of the effect of changing age on outcomes in childhood disease. Unlike in adults, this approach is critical for adolescence where there are rapid developmental changes with increasing age, which substantially impact outcomes. These are not taken into account in most studies to date, as age is not usually updated in theses cohort analyses [5,25,12–14].

Our study has several limitations. The study used retrospective routinely collected data, possibly misclassifying death as LTFU. A high proportion of CD4 counts at ART initiation was missing. This meant that the analysis did not take the degree of immunodeficiency into account. Adjustment for WHO stage made no substantive difference to the results (data not shown). The high proportion of missing CD4 counts was likely due to a combination of CD4 counts not being performed and not recorded in a routine health programme from which these data were obtained. Data on mode of infection were not available. However, gender was relatively balanced across age groups suggesting perinatal infection as the most likely route of infection for most of these children. Among the older adolescents, some might have been infected sexually, as there is a slight increase in the proportion of girls (56%) among the 15–19 year olds. The data were censored at the end of 2010 and thus might be considered “outdated”. However, Zimbabwe has had a much earlier onset and severe HIV epidemic with antenatal HIV prevalence peaking in 1997 [27]. As a result, large numbers of adolescents were already on ART in Zimbabwe during the period of this analysis (2004–2011, 5). Therefore, the findings have strong relevance to other countries with later onset epidemics, which are only now seeing comparable numbers of older children and adolescents who have grown up with HIV infection presenting to healthcare services. CD4 thresholds have changed over the last decade, with the most recent 2015 WHO guidelines recommending treatment of all HIV-infected individuals regardless of CD4 cell counts [28]. This policy will hopefully change the risks of morbidity and mortality in children and adolescents starting on ART now and in the future. Whether early start of ART changes the risk of LTFU remains to be seen [29,30]. Increasing numbers of HIV-infected children are surviving into adolescence and beyond making these findings and the novel analysis approach highly relevant for 2017.

To our knowledge, this is the first age-updated analysis investigating critical periods of transition across the age spectrum of 5–19 year olds using narrow five-year age-bands. We recommend this as an approach to investigate outcomes in children and adolescents with chronic disease, including HIV. Standardized analysis, taking into account the heterogeneity within children and adolescents, will facilitate high-quality studies. Older adolescents are at increased risk of LTFU even when they reach adulthood and importantly adolescents are the only age group in which HIV-related mortality is still rising [31]. Therefore, interventions specifically targeted at this age group are a high priority [3,32]. Thus far few evidence-based interventions exist targeting HIV-infected adolescents [33]. Systematic reviews assessing intervention among children and adolescents with other chronic diseases have also highlighted the need for more data and better quality studies [7,34,35]. Standardized and widely agreed methodology will foster high-quality research and add weight to the evidence to focus on adolescents living with HIV.

In summary, this study shows an increased risk of attrition of HIV-infected older adolescents and underscores the importance of accounting for changing in age in the analysis.

## Notes

To access the supplementary material to this article please see Supplementary Files under Article Tools online.
